# Supplementary material for: A cryptic pocket in Ebola VP35 allosterically controls RNA binding
Source: Nat Commun. 2022 Apr 27;13:2269. doi: 10.1038/s41467-022-29927-9 (PMC9046395; doi:10.1038/s41467-022-29927-9)
Supplement: Supplementary file 3 — Description of Additional Supplementary Files [file 41467_2022_29927_MOESM3_ESM.pdf]

Description of Additional Supplementary Files

File Name: Supplementary Movie 1
Description: A morph between the x-ray structure and an example open state of the blue exposon showing the large motion of helix 5 that opens the VP35 IID cryptic pocket. The movement of the helix on the beta sheet subdomain away from the alpha helix subdomain comprises pocket opening which the arrow highlights.

File Name: Supplementary Movie 2
Description: A morph between the x-ray structure and an example open state of the orange exposon showing the motion that gives rise to this exposon. The labels note the location of the N-terminus, and the two subdomains, the shown arrow highlights the movement of loops that comprise the orange exposon.
