# Supplementary material for: A cryptic pocket in Ebola VP35 allosterically controls RNA binding
Source: Nat Commun. 2022 Apr 27;13:2269. doi: 10.1038/s41467-022-29927-9 (PMC9046395; doi:10.1038/s41467-022-29927-9)
Supplement: Supplementary file 6 — Reporting Summary [file 41467_2022_29927_MOESM6_ESM.pdf]

nature portfolio

Corresponding author(s): Gregory R. Bowman

Last updated by author(s): Mar 28, 2022

Reporting Summary

Nature Portfolio wishes to improve the reproducibility of the work that we publish. This form provides structure for consistency and transparency in reporting. For further information on Nature Portfolio policies, see our Editorial Policies and the Editorial Policy Checklist.

Statistics

For all statistical analyses, confirm that the following items are present in the figure legend, table legend, main text, or Methods section.

n/a	Confirmed	
☐	☒	The exact sample size (*n*) for each experimental group/condition, given as a discrete number and unit of measurement
☐	☒	A statement on whether measurements were taken from distinct samples or whether the same sample was measured repeatedly
☒	☐	The statistical test(s) used AND whether they are one- or two-sided<br>*Only common tests should be described solely by name; describe more complex techniques in the Methods section.*
☒	☐	A description of all covariates tested
☐	☒	A description of any assumptions or corrections, such as tests of normality and adjustment for multiple comparisons
☐	☒	A full description of the statistical parameters including central tendency (e.g. means) or other basic estimates (e.g. regression coefficient) AND variation (e.g. standard deviation) or associated estimates of uncertainty (e.g. confidence intervals)
☒	☐	For null hypothesis testing, the test statistic (e.g. *F*, *t*, *r*) with confidence intervals, effect sizes, degrees of freedom and *P* value noted<br>*Give P values as exact values whenever suitable.*
☒	☐	For Bayesian analysis, information on the choice of priors and Markov chain Monte Carlo settings
☒	☐	For hierarchical and complex designs, identification of the appropriate level for tests and full reporting of outcomes
☒	☐	Estimates of effect sizes (e.g. Cohen's *d*, Pearson's *r*), indicating how they were calculated

Our web collection on statistics for biologists contains articles on many of the points above.

Software and code

Policy information about availability of computer code

Data collection	All molecular dynamics data were generated using Gromacs 5. with the AMBER03 forcefield with TIP3P solvent.
Data analysis	All reported data analysis was conducted using open-source python libraries and software including FAST, Exposons, Enspara, CARDS, DiffNets, MdTraj (and Shrake-Rupley Algorithm) 1.8.0, PyMol 2.x, Scipy 1.3.2, NumPy 1.14.x and 1.19.5, Matplotlib 3.5, Pandas 0.25.3, and Seaborn 0.11.2 FAST, Exposons, CARDS, and Diffnets are available at https://github.com/bowman-lab

For manuscripts utilizing custom algorithms or software that are central to the research but not yet described in published literature, software must be made available to editors and reviewers. We strongly encourage code deposition in a community repository (e.g. GitHub). See the Nature Portfolio guidelines for submitting code & software for further information.

Data

Policy information about availability of data

All manuscripts must include a data availability statement. This statement should provide the following information, where applicable:

- Accession codes, unique identifiers, or web links for publicly available datasets
- A description of any restrictions on data availability
- For clinical datasets or third party data, please ensure that the statement adheres to our policy

The molecular dynamics datasets generated during and analyzed in the current study are available from the corresponding author on reasonable request. MD start files are available with the MSM data in the below linked repository. Any request may take multiple business days to fulfill. Once shared, we will not enforce any limitations for how the data may be used. The MSM data and MD starting structures have been deposited in the Open Science Framework database https://osf.io/5pg2a. Data shown in graphs are available in the source data file. Referenced structures: PDB ID 3FKE [http://doi.org/10.2210/pdb3FKE/pdb] and PDB 3L26

March 2021

[http://doi.org/10.2210/pdb3L26/pdb].

Field-specific reporting

Please select the one below that is the best fit for your research. If you are not sure, read the appropriate sections before making your selection.

- [x] Life sciences
- [ ] Behavioural & social sciences
- [ ] Ecological, evolutionary & environmental sciences

For a reference copy of the document with all sections, see nature.com/documents/nr-reporting-summary-flat.pdf

Life sciences study design

All studies must disclose on these points even when the disclosure is negative.

Sample size	For the small system described, excess of 100 microseconds of MD simulation should be sufficient to observe meaningful dynamics and calculate the probabilities of the various conformational states the system adopts especially given the wide range of conformational states used to seed the Folding@Home simulations. Approximately 6.1 million Folding@home trajectory frames sums to approximately 122 microseconds of simulations were clustered as described resulting in 4469 states. Every frame in our simulations were used in the Exposons, CARDS and DiffNet analyses while the F239 Chi1 data comes from the MSM. Experimental sample sizes were preset to three unless otherwise needed due to technical problems with the experiment Final sample size is reported in each figure legend.
Data exclusions	For the F239 rotamer distribution, trajectories were randomly selected with replacement to fit a new MSM to calculate the standard deviation of the equilibrium probabilities which can allow for MD trajectories to be excluded. This was done to resample the equilibrium probabilities in the fit MSM. For binding and thiol-labeling experiments, no data were excluded.
Replication	MD simulation parameters are reported, we expect that repeating these simulations for sufficient sampling time under similar conditions should replicate our findings. Our MSM data has been made available as described in the Data Availability statement to facilitate recreating our analyses. Experimental data were reproducible from at least two separate rounds of recombinant protein purification and three replicates. Methods and Supplemental Information are provided in detail to aid the repeatability of our experiments and analyses.
Randomization	None, randomization was not appropriate in this study.
Blinding	None, blinding was not appropriate in this study.

Reporting for specific materials, systems and methods

We require information from authors about some types of materials, experimental systems and methods used in many studies. Here, indicate whether each material, system or method listed is relevant to your study. If you are not sure if a list item applies to your research, read the appropriate section before selecting a response.

Materials & experimental systems

n/a	Involved in the study	
x		Antibodies
x		Eukaryotic cell lines
x		Palaeontology and archaeology
x		Animals and other organisms
x		Human research participants
x		Clinical data
x		Dual use research of concern

Methods

n/a	Involved in the study	
x		ChIP-seq
x		Flow cytometry
x		MRI-based neuroimaging
